# Nutritional aspects in chronic non-cancer pain: A systematic review

**DOI:** 10.3389/fnut.2022.931090

**Published:** 2022-08-08

**Authors:** Inmaculada Xu Lou, Eugenia Gil-García, Rocío Cáceres-Matos, Kamran Ali, Esther Molina

**Affiliations:** ^1^Department of Nursing, Faculty of Nursing, Physiotherapy and Podiatry, University of Seville, Seville, Spain; ^2^International Education College of Zhejiang Chinese Medical University, Hangzhou, China; ^3^Department of Nursing, Faculty of Health Sciences, University of Granada, Granada, Spain; ^4^Biomedical Research Centre (CIBM), Institute of Neurosciences “Federico Olóriz,” University of Granada, Granada, Spain

**Keywords:** chronic pain, diet, feeding, nutrients, nutrition, supplements

## Abstract

**Objectives:**

Chronic pain (CP) is an unpleasant emotional and sensory experience that can be accompanied by tissue damage that persists for more than 3 months. Recent studies show that certain nutritional strategies can help to improve pain, so this study is aimed to systematically review scientific evidence to understand and map the effect of the use of nutritional strategies on the presence or intensity of chronic non-cancer pain (CNCP) and the association of these nutritional aspects with the presence or intensity of CNCP.

**Study design:**

A systematic review.

**Methods:**

Two independent researchers searched for randomized clinical trials (RCTs) and observational studies that explored the relationship between nutrition and CNCP in adults from 2010 to 2020 in PubMed, Web of Science, Scopus, and Cochrane Library databases. A total of 24 studies were included, of which 20 were RCTs and 4 were observational studies. They are classified into the administration of nutritional supplements, dietary modification, and incorporation of food.

**Results:**

Of these studies, those that have a significant effect on pain are dietary modification and the use of nutritional supplements. On the other hand, the main results from the few observational studies included in this review point to the existence of an association relationship between less pain and a ketogenic or hypocaloric diet or adherence to the Mediterranean diet.

**Conclusion:**

Dietary modification seems to be one plausible therapeutic option to improve and relieve CNCP. However, more research is needed in this regard to obtain better conclusions.

**Systematic Review Registration:**

[www.crd.york.ac.uk/prospero], identifier [CRD42021226431].

## Introduction

According to the International Association for the Study of Pain, chronic pain (CP) is defined as an unpleasant emotional and sensory experience that may or may not be accompanied by tissue damage that persists for more than 3 months (1, 2). When pain is not a consequence of an oncological process, it is called chronic non-cancer pain (CNCP) (3).

It is estimated that one in five people in the world suffers from CP and one in three cannot maintain an independent lifestyle due to pain (4).

It produces consequences in the performance of daily activities, in the practice of physical exercise (3), and poor quality sleep (5), and it is difficult to participate in social activities (6) with significant social and health costs (1, 7).

The main intervention for CP relief is the use of antalgic drugs, which gives rise to numerous adverse effects (7). Nevertheless, there are currently other approaches to pain, such as psychosocial strategies, physical activity interventions (2), or nutritional care (8), which seem to show positive results in pain relief.

Recent studies show the potential use of nutritional strategies to decrease pain sensation or reduce the risk of suffering from CP since it is cheaper than analgesic drugs and is less likely to produce adverse effects. That is why some researchers have tried to shed light on the role of nutritional elements in CP. Thus, our objective was to systematically review scientific evidence based on clinical and observational studies to understand and map the effect of the use of different nutrients, foods, or food supplements on the presence or intensity of CNCP, and the association of these nutritional aspects with the presence or intensity of CNCP.

## Materials and methods

### Search strategy and data sources

Between March and April 2020, a search was carried out for documents published in the last 10 years in the PubMed, Web of Science, Scopus, and Cochrane Library databases.

The search equation was as follows: (diet OR antioxidants OR micronutrients OR nutrition OR “integrative pain medicine” OR healing OR eating OR “nutritional status” OR “anti-inflammatory diet” OR food OR eating OR appetite OR “food habits” OR “food preferences” OR nutrient OR “diet therapy”) AND (“chronic pain” OR “persistent pain” OR “long term pain” OR pain OR “back pain” OR neuralgia OR “trigeminal neuralgia” OR hyperalgesia OR fibromyalgia OR “phantom limb” OR “complex regional pain syndromes” OR “nociceptive pain” OR headache OR endometriosis OR migraine OR arthritis) NOT (cancer OR tumor OR oncolog*).

### Inclusion criteria

The selected documents were (1) original articles or systematic reviews that explored the relationship between nutrition and CNCP; (2) published between 2010 and 2020; (3) in English or Spanish; (4) with experimental (randomized clinical trials; RCTs) or observational epidemiological design; (5) implemented in over 18 years old population, men, and/or women; (6) full text available, and (7) with sufficient methodological quality. Specifically, only those observational studies that had a high or acceptable methodological quality according to the Scottish Intercollegiate Guidelines Network (SIGN) tool (9) and experimental studies with a score greater than 3 on the Jadad scale (10) were included in the present review.

### Exclusion criteria

The exclusion criteria were (1) documents that studied pharmacological and surgical treatments with no nutritional approach for CNCP, (2) acute pain, and (3) as this systematic review is focused only on nutritional interventions, pain derived from surgical interventions or oncological processes was also excluded.

The search and screening of documents were carried out by two researchers independently and the discrepancies regarding the selected documents were resolved by consensus of the researchers. Registration was made in the International Prospective Register of Systematic Reviews (PROSPERO) with the code CRD42021226431.

A data extraction table was created for the documents included in the review (Table 1), with the following items: first author, year, type of pain, objectives, method, sample, duration, measuring instruments, intervention design, and results.

**TABLE 1:** Main characteristics of the studies included in this systematic review.

First author et. al./type of pain	Objectives	Method/Sample/Duration	Measuring instruments	Intervention design	Results
Abbasnezhad et al. (11) Irritable bowel syndrome (IBS)	To explore the effects of vitamin D supplementation on symptoms, severity score, and quality of life in patients with IBS	RCT *N* = 90 6 months	DS, IBS, VAS	50,000IU vitamin D_3_ (*n* = 45) Placebo (*n* = 45)	IBS symptoms improved in the two groups. Abdominal pain significantly improved in the vitamin D group (*p* < 0.007).
Cordero et al. (20) Fibromyalgia (FM)	To evaluate the effect of CoQ_10_ on clinical symptoms in FM patients.	RCT *N* = 20 70 days	FIQ, VAS	CoQ_10_ 300mg/day (*n* = 10) Placebo (*n* = 10).	Reduction in pain in CoQ_10_ compared to placebo (56%) and reduction in tender joints (44%) (*p* < 0.01).
Sawaddiruk et al. (21) FM	To study whether supplementing CoQ_10_ with pregabalin can reduce pain better than pregabalin alone in FM patients.	RCT *N* = 11 40 days	FIQ, VAS, Pressure Pain Limit	300mg/day CoQ_10_+150mg/day pregabalin. Placebo	VAS and FIQ decreased in CoQ_10_ compared to placebo (*p* < 0.05). Pregabalin + CoQ_10_ reduced pain more than placebo.
Dunn-Lewis et al. (13) OA	To examine the effect of multinutrient supplementation on physical capacity, fatigue, mood, and other factors in active men and women of ages 40-70.	RCT *N* = 31 63 days	PROMIS-57, Lequesne Knee Index, KOOS	0.25mg vitamin B_12_ + 6mg vitamin B_6_ + 0.40mg folic acid + 20mg pantothenic acid + 500mg taurine + 2000mg leucine + 500mg isoleucine + 500mg valine + 50mg green tea	Men show improvement in fatigue, pain and joint pain, although it does not occur in women.
Fukumitsu et al. (17) OA	To investigate the effect of administering olive extract containing maslinic acid (MA) over a 12-week period in elderly patients with mild knee joint pain, especially when climbing stairs.	RCT *N* = 20 12 weeks	VAS, SF-8	50 mg MA (*n* = 12) Placebo (n = 8)	Pain VAS does not change between the two groups (*p* = 0.65).
Malek et al. (18) OA	To assess the anti-inflammatory effects of L-carnitine supplementation in women with knee OA.	RCT *N* = 72 women 8 weeks	DS, VAS	750 mg L-carnitine tartrate (*n* = 36) Placebo (*n* = 36)	Difference in pain severity according to VAS (*p* < 0.05).
Rondanelli et al. (19) OA	To investigate the short-term anti-inflammatory and anti-pain potential of non-animal chondroitin sulfate (CS) supplementation in obese patients with OA.	RCT *N* = 60 12 weeks	VAS, WOMAC, SF36	600 mg Chondroitin sulfate/day (*n* = 30) Placebo (*n* = 30).	Improvement in WOMAC and VAS in CS in both knees (*p* = 0.001)
Shell et al. (14) OA	To examine the efficacy and tolerability of theramine (AAB) in patients with chronic back pain compared to or in combination with ibuprofen.	RCT *N* = 122 28 days	VAS, Roland-Morris Disability Questionnaire (RMDQ), Oswestry Low Back Pain Scale (OLBPS)	Ibuprofen 400mg-day Theramine 710mg/day Ibuprofen 400mg/day + theramine 10mg/day	In AAB group and the combined group there was significant improvement. In the AAB group, the RMDQ decreased by 50.3% and in the OLBPS, by 41.91%.
Ghavipour et al. (15) RA	To investigate the effect of POMx on disease activity and biomarkers of inflammation in patients with rheumatoid arthritis (RA).	RCT *N* = 55 8 weeks	DAS28, VAS, FCFQ	POMx (250mg/day with a concentration of 40% ellagicacid) (n = 30) placebo (n = 25)	Reduction of DAS28 score (*p* < 0.001), related to decrease in swelling (*p* < 0.001), tender joint count (*p* = 0.001), pain intensity (*p* = 0.003).
Helli et al. (16) RA	To examine the effect of sesamin on inflammatory markers and clinical indices in patients with RA.	RCT *N* = 44 women 6 weeks	DS, DAS28, VAS	200mg sesamin/day (*n* = 22) Placebo (*n* = 22)	Reduction of the number of tender joints and severity of pain compared to placebo (*p* < 0.05).
Santanam et al. (22) Endometriosis	To investigate whether the administration of antioxidants in patients with endometriosis can affect pelvic pain in women.	RCT *N* = 59 women 8 weeks	VAS	Vitamin E 1200IU + vitamin C 1000mg (*n* = 46) Placebo (*n* = 13)	Improvement of dysmenorrhea in antioxidant group (37%). Chronic pelvic pain improved in 43%.
Singh et al. (12) Pancreatitis	To evaluate the effect of antioxidant supplementation compared with placebo on pain and quality of life.	RCT *N* = 107 6 months	VAS	600μg selenium, 0.54g vitamin C, 9000IU b-carotene, 270IU vitamin E and 2g methionine (n = 54) Placebo (*n* = 53)	Reduction of pain intensity with VAS in both groups (*p* < 0.05).
Schell et al. (30) OA	To examine the effect of dehydrated strawberries on pain and biomarkers of inflammation in obese adults with knee OA.	RCT *N* = 17 26 weeks	ICOAP, HAQ, VAS, DS	50g dehydrated strawberries Placebo	Pain score and HAQ are lower in strawberries. Knee pain and total pain, using ICOAP, lower in strawberries (*p* < 0.05). No differences in VAS.
Schumacher et al. (28) OA	To evaluate the effect of cherry juice on the improvement of knee OA.	RCT *N* = 59 13 weeks	WOMAC	470 ml/day Cherry juice (*n* = 27) Placebo (*n* = 32)	WOMAC improvement (*p* = 0.002) and pain (*p* = 0.042) in cherry juice.
Hashempur et al. (29) OA	To evaluate the efficacy of green tea extract in patients with knee OA.	RCT *N* = 50 1 month	VAS, WOMAC	Green tea 1,500 mg/day + diclofenac 100 mg/day (*n* = 25) Diclofenac (*n* = 25)	Improvement in knee pain, functional capacity and joint stiffness in green tea group. VAS (*p* = 0.038).
Lindqvist et al. (25) RA	To investigate whether a diet rich in mussels, together with additional treatment, can reduce pathological activity in patients with RA.	RCT *N* = 39 30 weeks	DS, DAS28, VAS, HAQ, SF36	75g/day mussels (*n* = 20) Control (*n* = 19)	No difference was observed between both groups.
Pirouzpanah et al. (26) RA	To study the possible beneficial effects of chamomile tea consumption on DAS-28, VAS and symptoms in patients with RA.	RCT *N* = 44 women 42 days	VAS, DS, DAS28	6g/day chamomile (*n* = 22) Placebo (*n* = 22)	Number of tender joints changed significantly (*p* = 0.000). DAS-28, number of swollen joints and VAS did not change.
Thimóteo et al. (27) RA	To evaluate the effects of cranberry juice on biomarkers of inflammation and pathological activity in patients with RA.	RCT N = 41 90 days	DAS28, VAS	500ml/day Cranberry juice (*n* = 23) Control (*n* = 18)	Reduction (*p* = 0.048) in the perception of pain with DAS28.
Messier et al. (24) OA	To compare the effects of diet + physical exercise, diet alone, or physical exercise alone on pain, function, mobility, quality of life in overweight and obese patients with knee OA.	RCT *N* = 454 18 months	WOMAC, SF-36	Diet + exercise Diet (hypocaloric, low in fat and high in vegetables) Exercise (1h/day, 3 days/week)	D + E greater decrease in pain, according to WOMAC, compared to E (*p* = 0.004) and D (*p* = 0.001).
Zamani et al. (23) RA	To determine the symbiotic supplementation effects on clinical and metabolic parameters of patients with RA.	RCT *N* = 54 8 weeks	DS, DAS28, VAS	Symbiotic Lactobacillus acidophilus, Lactobacillus casei and Bifidobacterium bifidum + 800 mg inulin (*n* = 27) Placebo (*n* = 27)	Improvement of DAS28 (*p* = 0.004) and VAS pain (*p* < 0.001).
Di-Lorenzo et al. (31) Migraine	Assess whether a ketogenic diet has an effect on the clinical parameters of migraine.	OS *N* = 96 women 6 months	Headache frequency	Ketogenic diet (*n* = 45) Hypocaloric diet (*n* = 51).	The number of days with headache, frequency of headache attacks and consumption of drugs for headaches decreased in both groups (*p* < 0.0001).
Lourdudoss et al. (34) RA	To investigate potential associations between dietary intake of fatty acids and different pain patterns after treatment with antirheumatic drugs in early RA.	OS *N* = 591 3 months	FCFQ, VAS, DAS28	Fatty acids omega 3 and omega 6	No statistically significant association.
Shmagel et al. (33) OA	To investigate the association between magnesium intake and knee pain score in a prospective cohort of patients with knee OA.	Cohort study *N* = 2548 48 months	FCFQ, WOMAC, KOOS	Magnesium	Patients with lower magnesium intake had worse WOMAC and KOOS than those with higher magnesium intake (*p* < 0.001).
Veronese et al. (32) OA	To observe if a high adherence to the Mediterranean diet pattern is associated with a lower frequency of pain, stiffness, disability and depression.	OS cohort *N* = 4470	FCFQ, SF-12, WOMAC	Mediterranean diet	Greater adherence to a Mediterranean diet had a lower score in WOMAC (*p* < 0.0001), less pain and disability. Lower adherence to vegetables had a worse score in SF-12 (*p* = 0.01).

Antiox, antioxidants; DAS28, Disease Activity Score 28; DS, dietary survey; FCFQ, food consumption frequency questionnaire; FIQ, Fibromyalgia Impact Questionnaire; HAQ, Health Assessment Questionnaire; ICOAP, Intermittent and Constant Osteoarthritis Pain; KOOS, Knee Injury and Osteoarthritis Outcome Score; OA, osteoarthritis; OS, observational study; PROMIS-57, Patient-Reported Outcomes Measurement Information System-57; RA, rheumatoid osteoarthritis; RCT, randomized clinical trial; SF-36, short form health survey-36; SF-8, Short Form Health Survey-8; VAS, visual analog scale; vit, vitamin; WOMAC, Western Ontario and McMaster Universities Osteoarthritis Index.

## Results

### Study characteristics

**FIGURE 1:**
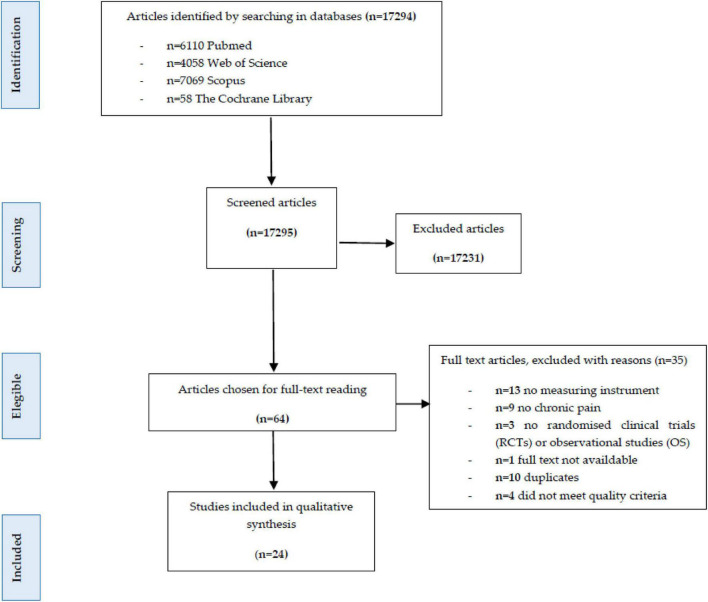
Preferred reporting items for systematic reviews (PRISMA) flow diagram.

A total of 17,295 documents were found. Of these, 64 articles were selected for full-text reading of which 24 documents were finally included. Figure 1 summarizes the selection process of the studies included in this review.

Regarding the epidemiological design of the studies included, 20 studies were experimental (RCTs), 2 studies were prospective cohort observational studies, 1 was the retrospective cohort, and 1 was case-control. The most common etiology of pain in the studies was osteoarthritis (*n* = 10), followed by rheumatoid arthritis (*n* = 7). Table 1 describes the main characteristics of the studies included in this systematic review.

### Main results from the experimental studies

The nutritional interventions evaluated for CNCP in the studies included the administration of nutritional supplements, dietary modification, and incorporation of food.

#### Administration of nutritional supplements

Regarding the studies carried out on pain caused by chronic pancreatitis, Abbasnezhad et al. (11) reported a significant improvement in pain during 6 months with the administration of 50,000 IU of vitamin D (*p* < 0.007). In addition, Singh et al. (12) described a significant reduction in the number of days with pain caused by chronic pancreatitis (*p* < 0.05) and a significant decrease in the intensity of the pain (*p* = 0.001) evaluated with the visual analog scale (VAS) after 3 months under treatment with an antioxidant compound of 600 μg of selenium, 0.54 g of vitamin C, 9,000 IU of beta carotene, 270 IU of vitamin E, and 2 g of methionine.

Regarding chronic back pain, Dunn-Lewis et al. (13) found a significant decrease (*p* < 0.05) in the intensity of back pain measured with the Patient-Reported Outcomes Measurement Information System–57 (PROMIS-57) and Knee Injury and Osteoarthritis Outcome Score (KOOS) instruments in men after supplementing the diet for 63 days with a multi-nutrient complex containing 0.25 mg of vitamin B_12_, 6 mg of vitamin B_6_, 0.40 mg of folic acid, 20 mg of pantothenic acid, 500 mg of taurine, 2,000 mg of leucine, 500 mg of isoleucine, 500 mg of valine, and 50 mg of green tea per supplement unit. However, no change in the intensity of pain was detected in women. Shell et al. (14) found a decrease in back pain intensity measured with the Roland Morris and Oswestry Disability Scales after 28 days of intervention with the combined administration of theramine (710 mg/day) and ibuprofen (*p* < 0.05).

Concerning patients with CP due to rheumatoid arthritis, Ghavipour et al. (15) supplemented the diet of the participants with two daily capsules of POMx (250 mg/day with a concentration of 40% ellagic acid) for 8 weeks and observed a significant reduction in rheumatoid arthritis pain perception measured with disease activity score-28 (DAS28; *p* < 0.001) and a decrease in the number of tender joints (*p* = 0.001) that also reduced pain intensity (*p* = 0.003). Helli et al. (16) observed that when 200 mg/day of sesamin was administered for 6 weeks, the number of tender joints and the intensity of pain evaluated with DAS28 and VAS were significantly reduced (*p* < 0.05 for both of them).

In the case of pain caused by osteoarthritis, Fukumitsu et al. (17) performed an intervention with maslinic acid with a dose of 50 mg/day for 12 weeks and found no significant difference in pain intensity measured with VAS when compared with the placebo group. However, Malek et al. (18), after using L-carnitine with a dose of 750 mg/day for 8 weeks, did find significantly lower pain intensity levels assessed by the VAS in the intervention group as compared to the control group (*p* = 0.019). Analogously, Rondanelli et al. (19) found that the Western Ontario and McMaster Universities Osteoarthritis Index (WOMAC) score in the group that had consumed chondroitin sulfate for 12 weeks at a dose of 600 mg/day had decreased significantly by 8.70 points, compared to the placebo group (*p* = 0.001).

On the other hand, Cordero et al. (20) evaluated pain in patients with fibromyalgia using Fibromyalgia Impact Questionnaire (FIQ). After the administration of 300 mg/day of CoQ_10_ for 70 days, they found a significant reduction in pain intensity (*p* < 0.01) and a significantly lower number of tender joints (*p* < 0.01) in comparison with the placebo. Furthermore, Sawaddiruk et al. (21) studied the effect of CoQ_10_ at a dose of 300 mg/day for 40 days in fibromyalgia and observed that the VAS and FIQ values decreased significantly in the CoQ_10_ group as compared to the placebo (*p* < 0.05).

Regarding dysmenorrhea, Santanam et al. (22) found a significant decrease in the number of painful days of the menstrual cycle in the group of participants who had ingested 1,200 IU of vitamin E and 1,000 mg of vitamin C for 8 weeks (*p* < 0.05). After the antioxidant intervention, chronic pelvic pain was decreased in 43% of the patients, and dysmenorrhea was descended in 37%.

For their part, Zamani et al. (23) carried out a clinical trial administering symbiotic supplements (Symbiotic *Lactobacillus acidophilus*, *Lactobacillus casei*, *Bifidobacterium bifidum*, and 800 mg inulin) for 8 weeks to patients with rheumatoid arthritis. A significant improvement was observed in scores measured with the DAS28 and VAS scales in this group (*p* = 0.004 and *p* < 0.001, respectively).

#### Dietary modification

In other research studies, diet intervention is accompanied by physical exercise. Messier et al. (24) carried out an intervention with a hypocaloric diet (low in fat and high in vegetables) combined with 1 h per day of physical training for 3 days a week, alternating aerobic and strength exercises in patients with osteoarthritis. The results showed that the compressive strength in the knee was decreased by 5% in the group that only did physical exercise (E), 10% in the group where only the diet was modified (D), and 9% in the diet group accompanied by physical exercise (D + E) at 18 months. However, in the D + E group, a greater decrease in pain was found, according to WOMAC, at 18 months when compared to E (*p* = 0.004) and D (*p* = 0.001).

#### Incorporation of food

The addition of foods, such as mussels, chamomile tea, blueberry or cherry juice, green tea, and strawberries, has been studied to evaluate the reduction of osteoarticular pain.

With respect to rheumatoid arthritis, Lindqvist et al. (25) observed that the group that consumed 75 g/day of mussels showed a lower intensity of perceived pain measured with DAS28 (*p* = 0.017). However, this difference was not observed when compared with the group that consumed meat (*p* = 0.200). Likewise, no statistically significant difference was obtained when comparing the number of tender joints and the assessment of pain intensity using the VAS tool when comparing the intervention group with the control group (*p* = 0.48). For their part, Pirouzpanah et al. (26) analyzed the effect of chamomile tea (6 g/day) on rheumatoid arthritis. The number of tender joints was decreased significantly (*p* < 0.001), although this change was not observed in the score measured by DAS28, the number of swollen joints, or the perception of pain. Regarding the consumption of blueberry juice (500 ml/day) at 90 days, Thimóteo et al. (27) stated that there was a significant reduction (*p* = 0.048) in the perception of pain when compared with the control group measured with the DAS28 instrument.

For osteoarthritis, Schumacher et al. (28) observed a significant improvement in the WOMAC score at 13 weeks in the group that consumed 470 ml/day of cherry juice (*p* = 0.002) when compared with the placebo group. The same relationship was observed by Hashempur et al. (29) in all the variables analyzed (knee pain, functional capacity, and joint stiffness) that included the VAS score (*p* = 0.038) in the group that consumed green tea (1,500 ml/day) during 30 days. In the control group, pain intensity only significantly descended when measured with WOMAC but not when measured with VAS.

Regarding knee pain, Schell et al. (30) described that in the group that consumed 50 g/day of strawberries, the intensity of pain was significantly lower at 12 weeks (*p* < 0.05) for both constant pain and intermittent pain, measured with the Intermittent and Constant Osteoarthritis Pain (ICOAP), although there were no differences in VAS for pain at the end of the 26 weeks of intervention.

### Main results of observational studies

Nutritional aspects, such as the type of diet or some supplements, have been evaluated from observational studies for their plausible relation to pain. Concerning diet modification studies, Di Lorenzo et al. (31) observed that the number of days with headache was decreased in the two groups that followed hypocaloric or ketogenic diet (*p* < 0.0001). However, this improvement had occurred earlier in the group with a hypocaloric diet, from the second month, while in the group with a ketogenic diet, it had occurred from the sixth month. On the other hand, other clinical variables, such as frequency of headache attacks or consumption of drugs for headaches, were decreased equally in the two groups from the sixth month (*p* < 0.0001). Furthermore, Veronese et al. (32) found that patients who had greater adherence to the Mediterranean diet had better scores in WOMAC (*p* < 0.0001) and less general pain evaluated by WOMAC (*p* < 0.05).

Regarding the observational studies about nutritional supplements, Shmagel et al. (33) focused on knee pain in patients with osteoarthritis and observed that a lower intake of magnesium in the diet was associated with worse scores on WOMAC and KOOS than those with higher magnesium intake (*p* < 0.001). Likewise, they found a relationship between people who had low magnesium intakes in their diet and greater intensity of knee pain due to osteoarthritis at 48 months of follow-up. However, Lourdudoss et al. (34) did not find a statistically significant association between the consumption of omega 3 fatty acids within the diet and pain due to rheumatoid arthritis nor did they found an association between supplementation with omega 3, omega 6, and the omega 6:omega 3 ratios with DAS28 scores.

## Discussion

The aim of this study was to review the scientific literature on the impact of the use of nutritional strategies among people with CNCP. We found that most of the interventions with nutritional supplements collected in our study show improvement and relief in CP (11, 13, 20, 21). This is also the case when it is modified to a hypocaloric, Mediterranean, or with a healthier profile diet (24, 31, 32). However, the use of stand-alone foods, such as fruit juices, yields few hopeful results (26, 30).

We found a few studies whose intervention was the modification of the diet, and it was easier to find studies whose intervention was by using a capsule or pill. This could be due to the ease of applicability of the second one, while the modification in diet requires more effort both in patients and researchers. That is why we understand the nutritional education of special relevance in these patients, highlighting above all the main difficulties they may go through, such as lack of knowledge, lack of interest, or rigidity in the face of change (35).

The use of nutritional interventions to relieve pain in clinical practice has numerous benefits, such as fewer adverse effects than drugs, being more economical methods, or increasing patient autonomy (7, 8, 36).

We observe that the intervention that offers the best results is diet modification. This is also confirmed by Brain et al. (8), Clinton et al. (37), and Kaartinen et al. (38). However, this modification has to be easy to wear, durable, and adapted to the patient to obtain the best results (35).

Brain et al. (8) included four types of interventions in their review, which were dietary modifications, nutrient intake modifications, use of nutritional supplements, and use of fasting. Comparing our systematic review with that carried out by Brain et al. (8), we found that their team did not include observational studies and interventions that were to add a specific food. In addition, they included non-RCTs, so we could find some bias. On the other hand, if we compare it with Ahmed Ali et al. (39), they conducted a systematic review that specifically focused on clinical trials on chronic pancreatitis, while our team has addressed a broader field.

The main limitation that we found in our study was that there are still a few studies on the relationship between nutrition and pain, maybe because it is a new topic (36). When comparing the 24 documents included in this review, the heterogeneity between them was revealed, which particularly affects the methodology and design of the intervention. It is for this reason that we could not do a meta-analysis. An effort is needed to carry out future research on this topic using validated instruments to assess non-cancer CP and the nutritional variables, with deep described homogeneous interventions on large and well-characterized patient samples.

## Conclusion

The results obtained show that there are nutritional interventions, especially diet modification, that can improve and alleviate CNCP. Furthermore, there is a need for future research to study CP as an independent entity and not as a symptom of the disease. If the evidence is strong, interventions could be applied in a clinical setting to improve the quality of life of patients suffering from this problem.

## Data availability statement

The original contributions presented in the study are included in the article/supplementary material, further inquiries can be directed to the corresponding author/s.

## Author contributions

IX obtained the data, performed the analysis, and contributed to writing the draft. EM performed the literature review, supervised all aspects of its implementation, contributed to the review of the manuscript, and contributed ideas and approved the final version. EG-G and RC-M supervised all aspects of its implementation, contributed to the revision of the manuscript, and contributed ideas and approved the final version. KA edited and corrected the article. All authors contributed to the article and approved the submitted version.
